# Occurrence and Characterization of *Sclerotinia sclerotiorum* Causing Fruit Rot on Sweet Cherry in Southern China

**DOI:** 10.3390/plants12244165

**Published:** 2023-12-15

**Authors:** Ruoxin Ruan, Kangkang Huang, Huifeng Luo, Chen Zhang, Dujun Xi, Jiabo Pei, Hui Liu

**Affiliations:** Institute of Horticulture, Hangzhou Academy of Agricultural Sciences, Hangzhou 310024, China; buffalo126@126.com (R.R.);

**Keywords:** *Sclerotinia sclerotiorum*, sweet cherry, fruit rot, pathogenecity, control, genome sequencing

## Abstract

Sweet cherry (*Prunus avium* L.) is widely planted in northern China due to its high economic value, and its cultivation has gradually spread south to warm regions. However, fruit rot, observed on the young fruits, poses a considerable threat to the development of sweet cherry. To determine the causal agent, morphological observation, molecular identification, and pathogenicity tests were performed on isolates obtained from diseased fruits. As a result, *Sclerotinia sclerotiorum* was identified as the pathogen. Pathogenicity tests on different sweet cherry cultivars indicated that ‘Summit’ was highly sensitive to *S. sclerotiorum*, whereas ‘Hongmi’ showed significant resistance. Besides sweet cherry, *S. sclerotiorum* could also infect other vegetable crops we tested, such as cowpea, soybean, tomato, and chili. Fungicide sensitivity and efficacy assays showed that both fludioxonil and pyraclostrobin can effectively inhibit the mycelial growth of *S. sclerotiorum* and decrease disease incidences on the young fruits of sweet cherry. Furthermore, genome sequencing resulted in a 37.8 Mb assembly of *S. sclerotiorum* strain ScSs1, showing abundant SNPs, InDels, and SVs with the genome of *S. sclerotiorum* reference strain 1980 UF-70. The above results provide an important basis for controlling the fruit rot of sweet cherry caused by *S. sclerotiorum* in China.

## 1. Introduction

Sweet cherry (*Prunus avium* L.) is considered to be a fruit crop with growing agronomic and economic importance worldwide [1,2,3]. The annual global production reached ~2.7 million tons in 2021 based on the data from the FAOSTAT database (fao.org/faostat). Sweet cherry was introduced to China in the 1870s and is mainly planted in the regions surrounding Bohai Bay [4,5]. Since its cultivation became widespread in China in the 1990s, the total harvested area has reached 8558 ha, with an annual production of 36,746.49 tons so far (FAOSTAT, 2021). The fruit of sweet cherry has bright colors, ranging from yellow to dark red, with a delicious flavor, and is also rich in nutrients, including a variety of anthocyanins, vitamins, phenolics, minerals, and dietary fibers [6,7,8,9,10]. Due to its popularity among consumers and high economic returns to growers, sweet cherry cultivation has expanded beyond the traditional regions in China in the past two decades [3,11,12,13]. Sub-optimal cultivation regions located in southern China, such as Hangzhou, have subtropical monsoon climates, for which a rainy spring is one of the climate characteristics. Therefore, the high humidity and suitable temperature in spring lead to high incidences of various fungal diseases in the orchards, such as leaf spot, gummosis, shoot blight, anthracnose, blossom blight, and fruit rot, which seriously restrict the sustainable development of the sweet cherry industry in these regions [2,14,15,16,17,18,19,20].

Fruit rots caused by various fungi are serious diseases of sweet cherry. They occur throughout the whole fruit development period, from the young fruit stage to the post-harvest stage. Several pathogenic fungi have been reported to cause fruit rot in California, Oregon, Maryland, of the USA, and Santiago, O’Higgins, Maule, of Chile, including *Monilinia fructicola*, *Botrytis cinerea*, *Sclerotinia sclerotiorum*, and *Alternaria alternata*, which are all important pathogens in agricultural production [14,21,22,23,24]. *B. cinerea* and *M. fructicola* were also identified to cause fruit rot in cherries in China [25,26]. Since sweet cherry was introduced into southern China, fruit rot has been one of the crucial risks hindering the development of sweet cherry production. In the absence of preventive measures, the incidence of fruit rot on young fruits reached about 10% in the field. Therefore, it is urgent to identify the specific pathogenic species and develop effective control strategies against fruit rot on sweet cherry in southern China.

A likely cause was *S. sclerotiorum* because it is a broad-host-range plant pathogen that can infect more than six hundred plant species [27,28]. Many of these plant species are economically important crops, including grains, oilseeds, vegetables, fruits, flowers, and weeds [29,30,31,32,33]. In this study, we aimed to identify the causal agent of sweet cherry fruit rot in southern China based on morphological characteristics, molecular identification, and pathogenicity tests, and investigate effective methods for the control of this disease. We also conducted genome sequencing to provide support for further studies on the pathogenesis mechanism of *S. sclerotiorum*.

## 2. Results

### 2.1. Isolation and Identification of Pathogen from Diseased Fruits of Sweet Cherry

**Figure 1:**
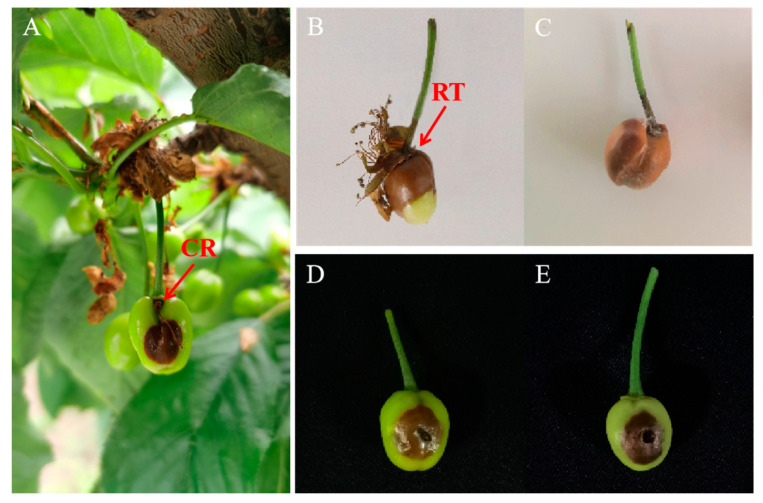
Symptoms of fruit rot on young fruits of sweet cherry. (**A**) Symptom on fruit of ‘Lapins’ in the field; diseased fruits of ‘Summit’ (**B**) and ‘Brooks’ (**C**) from the field; infected ‘Brooks’ fruits inoculated with isolates ScSs1 (**D**) and ScSs2 (**E**). RT and CR indicate receptacle and cracking region, respectively.

Fruit rot was observed on young fruits of sweet cherry during April in Hangzhou, China, including cultivars of ‘Lapins’, ‘Summit’, and ‘Brooks’ (Figure 1A). The symptoms started from the fruit receptacles or the cracking regions. The lesions with a light brown color extended to the fruit tops and stalks gradually (Figure 1B). A few days later, the entire fruit turned brown with soft rot and white fungal mycelium began to grow on the surface of the rotting fruits (Figure 1C). Eight diseased fruits were collected for isolation and purification of the pathogen that caused fruit rot. Based on preliminary *ITS* sequencing, we obtained three *B. cinerea* isolates, two *S. sclerotiorum* isolates, and three *Nigrospora* sp. isolates which were considered endogenous. Since *S. sclerotiorum* has not been reported on sweet cherry in China, we chose the *S. sclerotiorum* isolates for further study. The two isolates caused the same symptoms when inoculated on young fruits of sweet cherry as that in the field, labeled as ScSs1 and ScSs2 (Figure 1D,E).

**Figure 2:**
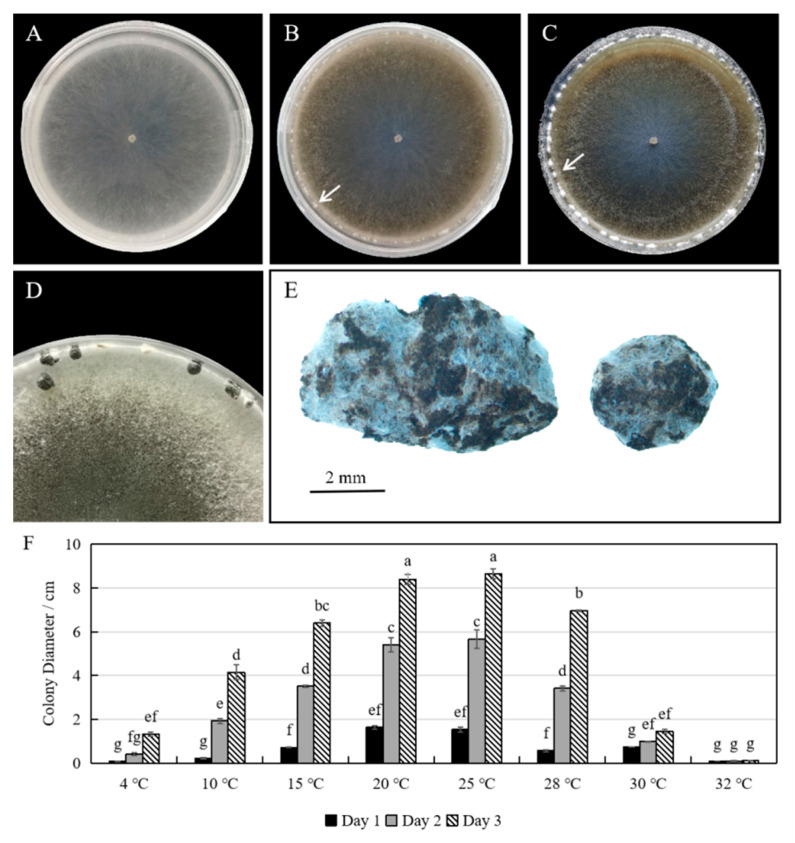
Colony morphology and sclerotium formation of isolate ScSs1. (**A**) Three-day-old colony; (**B**) six-day-old colony; (**C**) ten-day-old colony; (**D**) sclerotia on three-week-old culture; (**E**) sclerotia of isolate ScSs1; (**F**) colony diameters on PDA medium at 4, 10, 15, 20, 25, 28, 30, and 32 °C. The white arrows indicate the formation of sclerotia. Different letters on the bars are used to mark statistically significant differences from one another, as determined by one-way ANOVA (*p* < 0.05).

Morphological observation showed that the white colonies grew rapidly on PDA plates in the first three days (Figure 2A). Then, the colonies turned gray and white clumps of mycelium formed at the edge of the colonies gradually (Figure 2B,C). After two weeks, the mycelium clumps turned into sclerotia, which were black, hard, and irregular in shape (Figure 2D). The sclerotia measured 1.2–4.1 × 1.4–7.8 mm (Figure 2E). According to the growth curve, mycelium grew fastest at the temperature range of 20–25 °C, but stopped growing above 32 °C (Figure 2F). The colonies had already spread throughout the 9-cm plates after 3 days at 20 or 25 °C. The morphological characteristics were consistent with previous descriptions of *S. sclerotiorum* [14,21].

**Figure 3:**
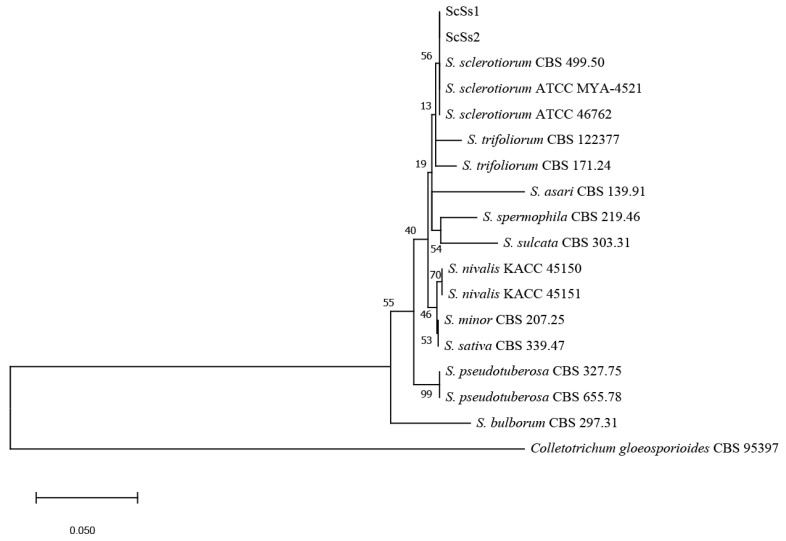
Phylogenetic analysis based on *ITS* sequences. The phylogenetic tree was inferred from the maximum likelihood method by MEGA-X. Bootstrap values supporting the branch are indicated at the nodes. The scale bar represents 0.05 nucleotide substitutions per site.

For molecular identification, the internal transcribed spacer (*ITS*) regions of isolates ScSs1 and ScSs2 were amplified and sequenced. The sequences were deposited in the GenBank (accession nos. OR578396 and OR578397 for ScSs1 and ScSs2, respectively). A maximum likelihood tree was conducted for phylogenetic analysis, including 15 other referred isolates of 10 *Sclerotinia* species and *Colletotrichum gloeosporioides* as an outgroup species (Table 1). The phylogenetic tree showed isolates ScSs1 and ScSs2 were both in the same clade as *S. sclerotiorum* (Figure 3). Isolates ScSs1 and ScSs2 were consistent in morphological and *ITS* sequences, so we chose ScSs1 as the subject of the subsequent experiments.

### 2.2. Pathogenicity Tests of S. sclerotiorum on Different Hosts

**Figure 4:**
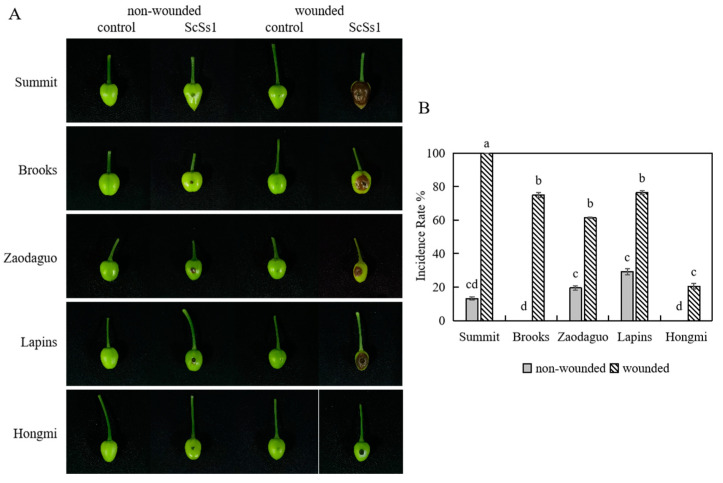
Pathogenicity tests performed on young fruits of sweet cherry. (**A**) Symptoms on inoculated young fruits; (**B**) incidence rates of diseased fruits. Different letters are used to mark statistically significant differences from one another, as determined by one-way ANOVA (F (df = 9) = 217.739; *p* < 0.001).

**Figure 5:**
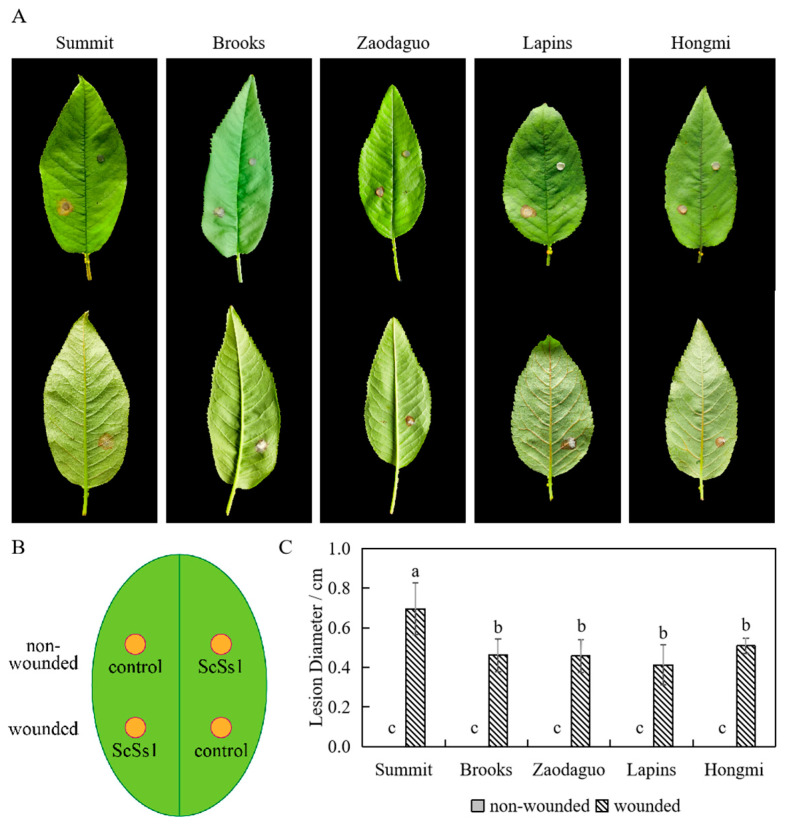
Pathogenicity tests performed on leaves of sweet cherry. (**A**) Symptoms on inoculated leaves. The top row is the front side of the leaves, the bottom row is the back side of the leaves. (**B**) Inoculation pattern diagram; (**C**) lesion diameters of diseased leaves. No lesions developed on non-wounded leaves. Different letters are used to mark statistically significant differences from one another, as determined by one-way ANOVA (F (df = 9) = 25.491; *p* < 0.001).

Pathogenicity tests were performed using the young fruits and leaves of five different cultivars of sweet cherry to verify the pathogenicity of *S. sclerotiorum* isolate ScSs1. On the young fruits, brown lesions were observed one day after inoculation and expanded rapidly (Figure 4A). Symptoms on leaves were observed at two days post-inoculation (Figure 5A). The incidence rates of non-wounded fruits were significantly lower than that of wounded fruits (Figure 4B). Intriguingly, *S. sclerotiorum* showed different pathogenicity on different sweet cherry cultivars. When inoculated non-wounded, young fruits of ‘Brooks’ and ‘Hongmi’ showed no symptoms, whereas ‘Summit’, ‘Zaodaguo’, and ‘Lapins’ had low incidence rates of fruit rot. When inoculated wounded, ‘Summit’ showed a 100% incidence rate of diseased fruits. ‘Brooks’, ‘Zaodaguo’, and ‘Lapins’ also displayed high incidence rates of fruit rot. However, ‘Hongmi’ showed a significantly lower incidence rate of fruit rot than other cultivars. Similar to the results on young fruits, tests on leaves also showed significant cultivar differences. Wounded inoculation rather than non-wounded inoculation could induce brown lesions on the leaves (Figure 5A). The lesion diameter on the leaves of ‘Summit’ was significantly larger than those of other cultivars. The causal agents were re-isolated from the lesions of the infected samples, and the colony morphology and molecular sequences were consistent with those of the original isolate ScSs1, which fulfilled Koch’s postulates.

**Figure 6:**
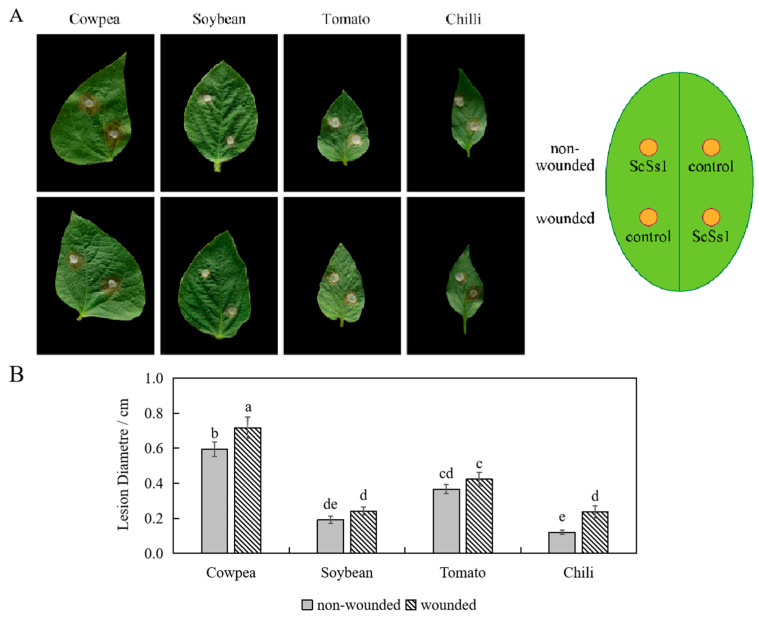
Pathogenicity tests performed on different crops. (**A**) Symptoms on inoculated leaves; (**B**) lesion diameters of diseased leaves. Different letters are used to mark statistically significant differences from one another, as determined by one-way ANOVA (F (df = 7) = 54.134; *p* < 0.001).

To verify the broad-host-range feature of *S. sclerotiorum*, crop species from Leguminosae and Solanaceae were chosen to perform the pathogenicity tests. Within two days, brown lesions spread across both the non-wounded and wounded inoculation sites, and the lesions on the wounded inoculation sites expanded more rapidly (Figure 6A). The average lesion diameters of inoculated leaves of cowpea were the largest, followed by those of tomato, soybean, and chili (Figure 6B). *S. sclerotiorum* isolates were successfully re-isolated from the inoculated leaves of cowpea, soybean, tomato, and chili.

### 2.3. Inibitory Effects of Fungicides against S. sclerotiorum

To effectively control fruit rot caused by *S. sclerotiorum*, we tested the fungicide sensitivity of 10 fungicides against isolate ScSs1 in vitro. The inhibitory effects are shown in Table 2. Most of these fungicides, except azoxystrobin, can effectively inhibit the mycelial growth of isolate ScSs1. Fludioxonil had the best inhibitory effect on ScSs1, with the lowest EC_50_ value of 0.02 ± 0.00 μg/mL. Carbendazim and pyraclostrobin also showed efficient inhibitory effects, with low EC_50_ values below 0.2 μg/mL, followed by chlorothalonil, procymidone, propiconazole, difenoconazole, tebuconazole, and boscalid in turn.

### 2.4. Control Effects of Fungicides on Sweet Cherry Fruit Rot Caused by S. sclerotiorum

The control effects of fludioxonil, carbendazim, and pyraclostrobin against *S. sclerotiorum* were assessed on young fruits of different sweet cherry cultivars (Table 3). The control effects ranged from 76.13% to 97.22% and 88.39% to 100.00% for 1 μg/mL fludioxonil and 1 μg/mL pyraclostrobin, respectively, on sensitive cultivars such as ‘Summit’, ‘Brooks’, and ‘Zaodaguo’. The fungicides tested were less effective on the relatively resistant cultivar ‘Hongmi’. None of these fungicides were effective on ‘Lapins’ at the tested concentrations. Although carbendazim can effectively inhibit the mycelial growth of *S. sclerotiorum*, it was not effective in controlling the fruit rot caused by *S. sclerotiorum*.

### 2.5. Genome Assembly and Variation Analysis of S. sclerotiorum Strain ScSs1

The assembled genome of *S. sclerotiorum* ScSs1 had a total size of 37.8 Mb, with a GC content of 41.6%. The genome completeness was estimated by BUSCO v4.0.2 (Benchmarking Universal Single-Copy Orthologs), showing that 98.2% of the BUSCOs were complete. In total, 10,874 protein-coding genes were predicted, with an average length of 1595 bp.

**Figure 7:**
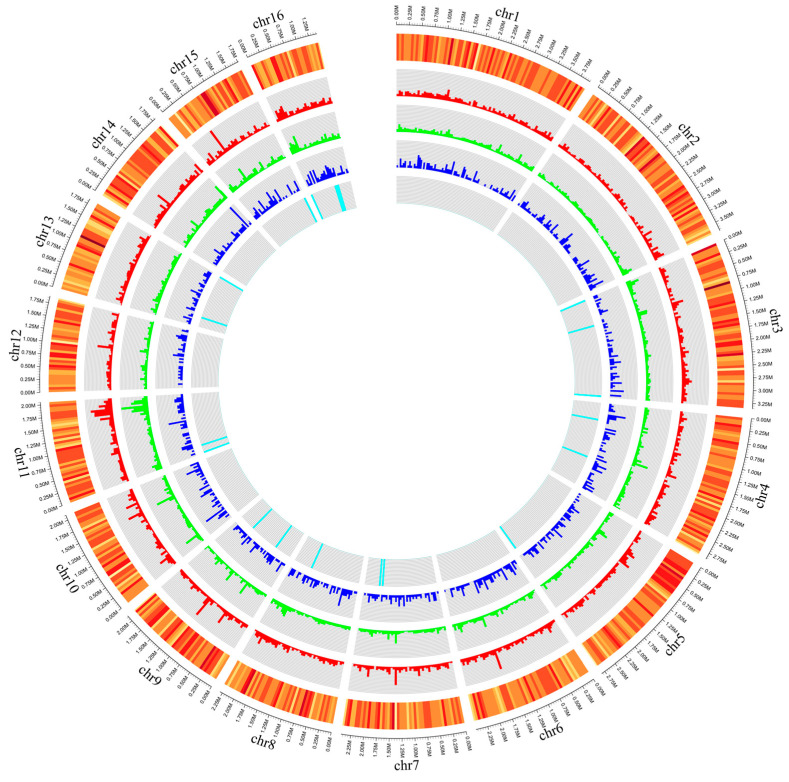
Circos plot depicting variation analysis of *S. sclerotiorum* strain ScSs1. The outermost circle shows chromosome size. Moving inward, the circles represent gene density (orange), SNPs (red), InDels (green), SVs (blue), and CNVs (cyan).

For comparative genomic analysis, the genome of *S. sclerotiorum* strain ScSs1 was compared with that of the *S. sclerotiorum* reference strain 1980 UF-70 (GenBank assembly accession: GCA_000146945.2), resulting in a mapped ratio of 94.63%. A total of 76,940 SNPs, 11,888 InDels, 1678 SVs, and 443 CNVs were identified in the variation analysis (Figure 7). Among the SNPs, 50,441 (33.08%) were intergenic, 11,061 (7.25%) were synonymous, and 11,061 (7.25%) were missense variants. In strain ScSs1, 5312 insertions and 5312 deletions of the InDels were found.

## 3. Discussion

Although regions of southern China belong to a sub-optimal cultivation area for sweet cherry, due to its popularity in the market and high economic benefits, more and more growers in these regions are trying to cultivate sweet cherry [11,13]. However, the diseases, owing to the rainy climate in spring, are critical factors affecting the development of sweet cherry in southern China. Fruit rot, observed on the young fruits of sweet cherry during April, has influenced sweet cherry cultivation in the Hangzhou region of southern China. Based on morphological characteristics and molecular identification, *S. sclerotiorum* was confirmed to be the causal agent of fruit rot on young fruits of sweet cherry in China. To our knowledge, this is the first report of *S. sclerotiorum* causing fruit rot on sweet cherry in southern China. Thus, it is necessary to study the characteristics and effective management of fruit rot caused by *S.sclerotiorum*.

*S. sclerotiorum* can cause diseases with different symptoms such as sclerotinia rot, stem rot, white mold, stem canker, shoot blight, blossom blight, crown rot, and fruit rot [34,35,36,37,38,39,40,41]. In Oregon, USA, and central Chile, it has been reported that *S. sclerotiorum* caused blossom blight and fruit rot on sweet cherry [14,21]. The sweet cherry cultivars ‘Lapins’, ‘Bing’, and ‘Staccato’ were verified to be susceptible to *S. sclerotiorum*. These reports provide a reliable basis for us to identify the causal agents of fruit rot on sweet cherry in China. Symptoms observed on the young fruits of sweet cherry in Hangzhou, China, were similar to those characterized in Oregon, USA [14]. In the pathogenicity tests, we also used fruits of ‘Lapins’ collected from Hangzhou, and the results also confirmed the previous report. Meanwhile, the *ITS* sequence of *S. sclerotiorum* isolated in Hangzhou shared a 100% identity with one of the previously deposited sequences (KF148608) from the fungus collected in central Chile [21,42]. The integrated characteristics confirmed *S. sclerotiorum* as the pathogen causing fruit rot on sweet cherry in China. Accordingly, frequent international fruit trade could be one of the routes for the rapid spread of pathogens [27].

*S. sclerotiorum* is known as a broad-host-range pathogen that infects hundreds of crops [27,31]. In this study, we found that *S. sclerotiorum* isolated from sweet cherry fruit can also form lesions on the leaves of other vegetable crops (Figure 6). Thus, the pathogen can easily spread from crops in the surrounding fields or environment to sweet cherry orchards under suitable conditions. Moreover, the optimal temperature range for the growth of isolate ScSs1 is 20–25 °C, which is similar to the range of the average daily temperature during April in Hangzhou. Early-to-mid-April is the stage of young fruit development in Hangzhou, and with rainy weather, young fruits at this stage are easily infected by *S. sclerotiorum.* In addition, we isolated *B. cinerea* from diseased young cherry fruits, which caused similar symptoms to *S. sclerotiorum*. *B. cinerea* is also an important plant pathogen known to cause gray mold disease on various hosts and has been reported to cause fruit rot in sweet cherry [21]. Therefore, it is necessary to prevent the development of those pathogens occuring in orchards and the surrounding environment in early spring in these regions.

The selection of resistant cultivars is one of the effective methods to resist diseases in agriculture production. The sweet cherry cultivars used in this study all performed well on physiological characteristics and fruit quality in sub-optimal cultivation regions of southern China, such as Shanghai and Hangzhou [43,44]. According to the results of pathogenicity tests on fruits and leaves, ‘Summit’ was the most sensitive to *S. sclerotiorum* among the five sweet cherry cultivars (Figure 4 and Figure 5). ‘Hongmi’ showed significant resistance to *S. sclerotiorum* in young fruit inoculation tests (Figure 4). ‘Hongmi’ is a sweet cherry cultivar bred in China with a yellowish-red color and it is resistant to cracking, which helps to defend against infection by pathogens [45]. These results could help growers select suitable resistant cultivars of sweet cherry in southern China.

The efficient and appropriate use of fungicides could prevent and control the diseases caused by pathogenic fungi. Carbendazim, a systemic benzimidazole carbamate fungicide, has been used to control *S. sclerotiorum* on oilseed rape (*Brassica napus*) in China [46,47]. In this study, carbendazim could significantly inhibit the mycelial growth of *S. sclerotiorum* (Table 2), but it was phytotoxic to sweet cherry fruits [48,49]. As a consequence, it is not recommended to be used for the control of diseases that happen during the fruit development stage. Fludioxonil, a phenylpyrrole fungicide, is widely applied to control post-harvest fungal diseases on cherry fruits and has already been registered to control gray mold on cherry in China [50]. Fludioxonil had the strongest inhibitory effect on the hyphal growth of *S. sclerotiorum* among the 10 fungicides we tested, and could effectively control symptoms of fruit rot on young fruits of sweet cherry (Table 2 and Table 3). As a fungicide belonging to the strobilurin group, pyraclostrobin has been registered for use on 57 categories of crops in China and is applied to control several fungal diseases, including gray mold, powdery mildew, brown rot, leaf spot, and anthracnose on fruit crops [51,52]. It is also labeled for application in cherry orchards during the bloom period in the USA [51]. In this study, pyraclostrobin significantly inhibited the development of *S. sclerotiorum* both in vitro and in vivo (Table 2 and Table 3). Consequently, both fludioxonil and pyraclostrobin can potentially be used to control fruit rot on sweet cherry caused by *S. sclerotiorum.* Since *S. sclerotiorum* may be a novel pathogen causing fruit rot on sweet cherry in southern China, the isolates were sensitive to most of the fungicides tested in this study, which provides more choices for chemical control against *S. sclerotiorum*.

For further studies on the infection mechanism and pathogen–host interaction of *S. sclerotiorum* on sweet cherry, we conducted genome sequencing of strain ScSs1 using the Illumina NovaSeq system. The genome size and GC content of this *S. sclerotiorum* strain were 37.8 Mb and 41.6%, respectively. The genome assembly features were similar to those of the reference strain 1980 UF-70 isolated from the USA, which was 38.9 Mb in size with a GC content of 41.6% [53,54]. The genome data and variation analysis of strain ScSs1 could also provide resources for research on the divergence of the *S. sclerotiorum* populations from various hosts and different regions.

## 4. Materials and Methods

### 4.1. Sample Collection and Fungus Isolation

Young fruits with fruit rot symptoms were collected from the sweet cherry orchard located in the Hangzhou Academy of Agricultural Sciences (30.16° N, 120.09° E), Hangzhou City, Zhejiang Province, China. Eight symptomatic fruits collected from fields were surface-sterilized in 75% ethanol for 30 s and rinsed three times with sterile distilled water. Small portions of 2 mm in diameter were excised from the margin between the lesioned and healthy tissues and placed on potato dextrose agar (PDA) supplemented with 100 μg/mL streptomycin sulphate. After incubation at 25 °C for 2 days, the hyphal tips of the fungal colonies were transferred to fresh PDA plates and incubated at 25 °C for further experiments.

### 4.2. Morphological and Biological Characterization

A preliminary identification was conducted based on colony morphology and sclerotia characteristics [34]. To observe colony morphology, 5-mm mycelial plugs taken from the edges of the actively growing cultures were placed on PDA and incubated at 25 °C under a 12 h photoperiod. The morphological features, including mycelial growth rate, color, and texture, of the colonies were recorded 3 days post-inoculation. Sclerotia formation was observed 10 days post-inoculation. Sclerotia were collected from each culture and measured for size, quality, and color after 3 weeks. To determine the optimum temperature range for the fungus, the growth rates of isolate ScSs1 were assessed on PDA medium at 4, 10, 15, 20, 25, 28, 30, and 32 °C. Five replicates were taken at each temperature. The colony diameters were measured daily and the growth curves were conducted after three days. The experiments were carried out in triplicates.

### 4.3. Molecular Identification and Phylogenetic Analysis

For molecular identification, fungal isolates were incubated on PDA plates for 5 days at 25 °C. The total genomic DNA from 100 mg of dried mycelia was extracted using an Easy Plant Genomic DNA Extraction Kit (Easy-Do Biotech, Hangzhou, China). The partial region of the internal transcribed spacer (*ITS*) was amplified with the primer pair *ITS1* and *ITS4* [35,36,42,55]. PCR amplification was conducted in a 20 μL reaction mixture containing 100 ng of genomic DNA, 10 µL 2 × Taq Plus Master Mix II, and 10 µM of each primer (Vazyme Biotech Co., Ltd., Nanjing, China). The PCR conditions were as follows: initial denaturation at 95 °C for 3 min; 35 cycles of denaturation at 95 °C for 15 s, annealing at 55 °C 15 s, and extension at 72 °C for 1 min; and a final extension at 72 °C for 1 min. The PCR products of the eight isolates were verified by 1% agarose gel electrophoresis and sequenced using Sanger sequencing by Sunya Biotechnology Co. Ltd. (Hangzhou, China). Phylogenetic analysis was performed using MEGA-X based on the maximum likelihood method with bootstrap values based on 1000 replications [56,57,58]. All the isolates used in the phylogenetic analysis are listed in Table 1 [34,37].

### 4.4. Pathogenicity Tests

The isolates were incubated on PDA plates at 25 °C for pathogenicity assessment. Ten-year-old sweet cherry trees were used to provide plant materials in this study, including cultivars ‘Summit’, ‘Brooks’, ‘Zaodaguo’, ‘Lapins’, and ‘Hongmi’, which were introduced in 2013 from the Dalian Academy of Agricultural Sciences, Dalian, China. Detached healthy sweet cherry fruits and leaves without injury were collected from the orchard in the Hangzhou Academy of Agricultural Sciences and washed under running tap water for 30 min and sterilized with 75% ethanol, then rinsed and air-dried. For host range determination, fresh leaves of cowpea, soybean, tomato, and chili were collected from vegetable fields surrounding the orchard and surface-sterilized as described above. Half of the disinfected samples were wounded with a bunch of 5 sterile needles and the other half were non-wounded. Both wounded and non-wounded samples were inoculated with 5-mm mycelial plugs taken from 3-day-old cultures. Negative controls were inoculated with sterile distilled water. Fifteen samples were included in each group. All the inoculations were maintained in a growth chamber at 25 °C under high humidity (>90%). Symptoms were scored at 2 dpi. The experiments were carried out in triplicates. The incidence rate was calculated as Equation (1):

Incidence rate (%) = 100 × Number of diseased fruits/Total number of fruits(1)

To fulfill Koch’s postulates, symptomatic tissues were excised from the diseased samples for re-isolation. The isolates were confirmed for identities with the original fungus based on morphological and molecular identification.

### 4.5. Fungicide Sensitivity Assays

To determine fungicides with a good control effect on *S. sclerotiorum*, we assessed 10 fungicides as follows: difenoconazole (10% active ingredient (a.i.); Syngenta Nantong Crop Protection Co., Ltd., Nantong, China), pyraclostrobin (25% a.i.; BASF Plant Protection Jiangsu Co., Ltd., Rudong, China), carbendazim (50% a.i.; Sichuan Runer Technology Co., Ltd., Chengdu, China), azoxystrobin (25% a.i.; Syngenta Nantong Crop Protection Co., Ltd., Nantong, China), procymidone (50% a.i.; Sumitomo Chemical Co., Ltd., Tokyo, Japan), chlorothalonil (75% a.i.; Syngenta Suzhou Crop Protection Co., Ltd., Suzhou, China), boscalid (50% a.i.; BASF SE, Ludwigshafen, Germany), fludioxonil (50% a.i.; Syngenta Suzhou Crop Protection Co., Ltd., Suzhou, China), tebuconazole (43% a.i.; Bayer Crop Science China Co., Ltd., Hangzhou, China), and propiconazole (25% a.i.; Syngenta Suzhou Crop Protection Co., Ltd., Suzhou, China). A 5-mm mycelial plug was placed on each PDA plate, supplemented with fungicides of different concentrations [59]. Each treatment contained three replicates and the entire experiment was repeated three times. The colony diameter (mm) was measured after 3 days of incubation. The inhibition rate of mycelial growth (Figure 4B) was calculated as Equation (2):

Inhibition rate (%) = 100 × ((Mycelial growth diameter of control colony − Mycelial growth diameter of treatment colony)/(Mycelial growth diameter of control colony − 5))(2)

EC_50_ (50% effective concentration) values were estimated statistically using IBM SPSS Statistics (SPSS Inc., Chicago, IL, USA).

### 4.6. Fungicide Effects on Controlling Fruit Rot Caused by S. sclerotiorum

Three fungicides were used in the control effect tests, fludioxonil, carbendazim, and pyraclostrobin. Control efficacy experiments were performed in an artificial climate chamber under the conditions of 25 °C light for 12 h and 20 °C darkness for 12 h. The fruits of ‘Summit’ and ‘Brooks’ were used to determine the optimal concentrations of each fungicide. The fruits of ‘Zaodaguo’, ‘Lapins’, and ‘Hongmi’ were then used to confirm the effect of each fungicide. Healthy sweet cherry fruits were surface-sterilized, as described in the pathogenicity tests. Then, 20 μL of different concentrations of fungicides was pipetted onto each fruit and air-dried [60]. Each fruit was wounded with sterile needles and inoculated with 5-mm mycelial plugs. Fifteen fruits were included in each group; the incubation conditions were described above in Section 4.4. The lesion diameters were measured at 2 dpi and the control effect was calculated as Equation (3). Data analysis was performed on IBM SPSS Statistics. Statistically significant differences were determined by one-way ANOVA (*p* < 0.05).

Control effect (%) = 100 × (Lesion diameter of control − Lesion diameter of treatment)/Lesion diameter of control(3)

### 4.7. Genomic Analysis

#### 4.7.1. Genomic DNA Library Preparation and Sequencing

Mycelia of *S. sclerotiorum* strain ScSs1 were cultured in potato dextrose broth (PDB) under a rotary shaker with 160 rpm at 25 °C for three days. Genomic DNA was extracted from mycelia using the CTAB method [59]. The quality of purified genomic DNA was verified by 1% agarose gels and the DNA concentration was measured by ND-2000 (NanoDropTechnologies, Wilmington, DE, USA). A total amount of 0.5 μg high-quality DNA (OD260/280 = 1.8~2.0) was used as input material for the DNA library preparations. The sequencing library was generated using a Truseq Nano DNA HT Sample Prep Kit (Illumina USA, San Diego, CA, USA) following the manufacturer’s recommendations. Briefly, the genomic DNA sample was fragmented by sonication to a size of 350 bp, then endpolished, A-tailed, and ligated with the full-length adapter, followed by further PCR amplification. After PCR products were purified AMPure XP kit (Beckman Coulter, Inc., Miami, FL, USA, AMPure XP system), the libraries were analyzed for size distribution by Agilent 2100 Bioanalyzer and quantified by real-time PCR (3 nM). The paired-end DNA-seq sequencing library was sequenced with the Illumina NovaSeq system at Shanghai Majorbio Bio-pharm Technology Co., Ltd. (Shanghai, China).

#### 4.7.2. Variant Discovery

Raw reads of low quality (mean phred score < 20), including reads containing adapter contamination and unrecognizable nucleotide (N base > 10), were trimmed or discarded by Fastp software (v0.23.4) [61]. Reads after trimming were mapped to their reference using BWA-MEME under default mapping parameters [62].

As the modified GATK best practices [63], the alignment bam files were sorted by SAMtools [64] and PCR duplicates were marked by MarkDuplicates. After performing the base quality recalibration, the variants containing SNPs (single nucleotide polymorphisms) and InDels (insertion-deletions) were called using the Haplotyper and GVCFtyper program in Sentieon Genomics Tools [65]. Variants were filtered using standard hard filtering parameters according to the GATK best practices pipeline.

SVs (structural variations) were reported by Manta, which detected structural variations based on several sources, including split alignments, soft clipping, insert size, paired alignments, and transchromosomal events [66]. CNVs (copy number variations) were detected by CNVkit, which used a method called the core window method to detect copy number variations [67]. All of the variants were annotated using SnpEff [68]. SNPs, InDels, and SVs were categorized based on their positions on the chromosome, including intergenic regions, exons, introns, splicing sites, untranslated regions, and 1-kb upstream and downstream regions, and their effects, including missense, start codon gain or loss, stop codon gain or loss, and splicing mutations.

## 5. Conclusions

In this study, *S. sclerotiorum* was determined as the causal agent of fruit rot on young fruits of sweet cherry in southern China. The local climate in spring is suitable for the growth of *S. sclerotiorum*; thus, the control of the disease will be a vital challenge for the sustainable development of sweet cherry production in this region. We screened five sweet cherry cultivars in the pathogenicity tests and found that ‘Summit’ was highly sensitive to *S. sclerotiorum*, whereas ‘Hongmi’ showed significant resistance inversely. Based on the results of toxicity tests and control effect tests, fludioxonil and pyraclostrobin could effectively control fruit rot on sweet cherry caused by *S. sclerotiorum.* Finally, genome data of the pathogenic strain ScSs1 will be a useful resource for future studies on the mechanism of the *S. sclerotiorum*–host interaction.

## Figures and Tables

**Table 1:** Descriptions and sequence accession numbers of reference isolates used in the phylogenetic study.

Species	Strain Accession	Origin	GenBank Accession of *ITS*
*Sclerotinia sclerotiorum*	CBS 499.50	Netherlands	MH856725
*Sclerotinia sclerotiorum*	ATCC MYA-4521	USA	FJ810516
*Sclerotinia sclerotiorum*	ATCC 46762	USA	JX648201
*Sclerotinia trifoliorum*	CBS 171.24	USA	MF964318
*Sclerotinia trifoliorum*	CBS 122377	Poland	KT970794
*Sclerotinia nivalis*	KACC 45150	Korea	HM746662
*Sclerotinia nivalis*	KACC 45151	Korea	HM746663
*Sclerotinia asari*	CBS 139.91	USA	MF964313
*Sclerotinia spermophila*	CBS 219.46	USA	MF964316
*Sclerotinia sulcata*	CBS 303.31	Netherlands	MH855222
*Sclerotinia pseudotuberosa*	CBS 327.75	Italy	AY526234
*Sclerotinia pseudotuberosa*	CBS 655.78	Italy	AY526231
*Sclerotinia minor*	CBS 207.25	Netherlands	MH854848
*Sclerotinia bulborum*	CBS 297.31	Netherlands	MH855218
*Sclerotinia sativa*	CBS 339.47	Netherlands	MH856278
*Colletotrichum gloeosporioides*	CBS 95397	Thailand	FJ972609

**Table 2:** Inhibitory effects of fungicides against *S. sclerotiorum*.

Fungicides	Regression Equation	EC_50_ (μg/mL)	Coefficient of Determination (R^2^)	95% Confidence Interval
Difenoconazole	y = 0.76 + 1.79x	0.38 ± 0.00	0.847	0.21~0.55
Pyraclostrobin	y = 0.84 + 0.92x	0.16 ± 0.03	0.891	0.00~0.43
Carbendazim	y = 1.75 + 1.89x	0.15 ± 0.00	0.942	0.10~0.24
Azoxystrobin	y = −1.12 + 1.54x	5.62 ± 0.07	0.954	3.34~7.74
Procymidone	y = 1.32 + 2.87x	0.36 ± 0.00	0.890	0.25~0.47
Chlorothalonil	y = 0.64 + 0.96x	0.32 ± 0.00	0.874	0.00~0.96
Boscalid	y = 0.76 + 1.79x	0.56 ± 0.05	0.915	0.05~1.43
Fludioxonil	y = 3.06 + 1.71x	0.02 ± 0.00	0.885	0.01~0.03
Tebuconazole	y = 0.64 + 1.73x	0.44 ± 0.02	0.952	0.08~0.77
Propiconazole	y = 0.84 + 1.88x	0.37 ± 0.01	0.935	0.25~0.51

x and y indicate the fungicide concentration and inhibition rate, respectively.

**Table 3:** Percentage control with fungicides against fruit rot on different sweet cherry cultivars.

Host Cultivars	Fludioxonil		Carbendazim		Pyraclostrobin	
	0.1 μg/mL	1 μg/mL	0.1 μg/mL	1 μg/mL	0.1 μg/mL	1 μg/mL
Summit	9.03 ± 1.70 ^a^	76.13 ± 1.25 ^d^	57.63 ± 6.89 ^c^	23.01 ± 5.73 ^ab^	17.42 ± 1.87 ^ab^	88.39 ± 2.99 ^de^
Brooks	50.83 ± 9.49 ^c^	96.68 ± 2.17 ^e^	17.32 ± 0.72 ^ab^	33.66 ± 4.42 ^b^	37.25 ± 5.17 ^b^	98.04 ± 1.88 ^e^
Zaodaguo	--	97.22 ± 2.62 ^e^	--	58.33 ± 4.90 ^c^	--	100.00 ± 0.00 ^e^
Lapins	--	11.03 ± 7.26 ^a^	--	5.67 ± 5.46 ^a^	--	6.90 ± 6.60 ^a^
Hongmi	--	39.25 ± 10.00 ^b^	--	7.88 ± 7.84 ^a^	--	47.81 ± 11.23 ^c^

Values indicate the mean ± SD of three replicates. Small letters indicate significant differences at 5% level (F (df = 20) = 24.849; *p* < 0.05).

## Data Availability

Data are contained within the article.
